# Fibrocytes: A Critical Review and Practical Guide

**DOI:** 10.3389/fimmu.2021.784401

**Published:** 2021-12-17

**Authors:** James W. Reinhardt, Christopher K. Breuer

**Affiliations:** ^1^ Center for Regenerative Medicine, Abigail Wexner Research Institute, Nationwide Children’s Hospital, Columbus, OH, United States; ^2^ Department of Surgery, The Ohio State University Wexner Medical Center, Columbus, OH, United States; ^3^ Department of Surgery, Nationwide Children’s Hospital, Columbus, OH, United States

**Keywords:** fibrocytes, collagen, myofibroblasts, bone marrow chimera, lineage tracing

## Abstract

Fibrocytes are hematopoietic-derived cells that directly contribute to tissue fibrosis by producing collagen following injury, during disease, and with aging. The lack of a fibrocyte-specific marker has led to the use of multiple strategies for identifying these cells *in vivo*. This review will detail how past studies were performed, report their findings, and discuss their strengths and limitations. The motivation is to identify opportunities for further investigation and promote the adoption of best practices during future study design.

## 1 Introduction

Fibrocytes are hematopoietic-derived cells that directly contribute to fibrosis of tissues throughout the body by producing collagen following injury, during disease, and with aging. While fibrocytes can be isolated from peripheral blood and enriched in culture, the lack of a fibrocyte-specific marker has challenged study of this cell type *in vivo*. One of the most common approaches has been to use bone marrow (BM) chimera models that enable tracking of hematopoietic-derived cells. However, by focusing on cells recently derived from the circulation some studies have overlooked hematopoietic-derived tissue resident populations. In addition, immunolabeling against type I collagen (Col1) has been performed to identify collagen expression without validation of expression by a secondary method. This is problematic because macrophages from which fibrocytes may be derived can degrade collagen and take up collagen fragments from their environment. Immunolabeling against Col1 only shows whether the protein is within the cell and does not distinguish whether this collagen has been produced by the cell or taken up (1–3). Therefore, on the one hand some studies may have under-identified fibrocytes, and on the other hand studies may have over-identified hematopoietic cells that produce collagen by falsely identifying macrophages with internalized collagen as fibrocytes. The field recognizes and has begun to address these limitations by using transgenic mouse models, immunolabeling against more robust targets of collagen production, such as collagen propeptides, and validating collagen expression by performing transcriptional analyses. Most recently, single-cell RNA sequencing (scRNA-Seq) has been applied to study macrophage fibroblastic differentiation and collagen production. This approach provides a broad and quantitative measurement of gene transcription, enhancing the ability to identify and characterize cells, thereby overcoming many limitations common among *in vivo* studies of fibrocytes.

In anticipation of a new era demarcated by the application of next generation sequencing and single-cell transcriptional analysis, now seems an appropriate moment to revisit what has been learned about fibrocytes and also how that knowledge was acquired to promote the application of best practices moving forward. This review will begin by detailing the methods that have been used to study fibrocytes including immunolabeling, transgenic mice, parabiosis models, and transcriptional analyses. Examples will be provided that cover several tissues throughout the body including skin, heart, lung, kidney, and liver. Through doing so, the intent is to familiarize the reader with the strengths and limitations of these methods as well as tissue-specific differences. Following next, efforts to quantify the relative amount of collagen production by fibrocytes, compared to traditional fibrogenic cells, and characterize fibrocyte-derived myofibroblasts will be summarized. Last, potential origins of fibrocytes and sites of fibrocyte differentiation will be discussed.

## 2 Background

Fibrocytes were first described in 1994 after observing spindle-shaped, adherent, fibroblast-like cells within subcutaneous wound chambers in mice (4). While fibroblasts are known to invade wound chambers from the surrounding tissue, these fibrocytes were observed acutely, within 2 days, coincident with the influx of peripheral blood cells and in an “*unexpectedly large number*”. The timing and abundance of these cells led the authors to conclude *“this cell population was arising from peripheral blood and not exclusively by slow migration from adjacent connective tissue”.* Cells isolated from the wound chamber were reported to have expressed Col1, as determined by immunolabeling, but lacked non-specific esterases indicating they were distinct from adherent peripheral blood monocytes. To follow up on the hypothesis that these fibrocytes were circulation-derived, human peripheral blood mononuclear cells (PBMCs) isolated by density gradient centrifugation were studied in tissue culture. Following plating, on the day these cells were isolated ~0.25% were Col1+ CD34+. Adherent cells were then cultured for 2-4 weeks in serum-supplemented medium under conditions that selected for plastic adherence. This resulted in enrichment of Col1+ CD34+ cells with an elongated morphology that also expressed the myeloid integrin CD11b as well as mesenchymal markers vimentin and fibronectin. These cells did not express epithelial, endothelial, or SMC markers, but did express leukocyte common antigen (CD45). When revisiting the wound chamber assay, 10 days after implantation 10-15% of cells in the wound chamber were Col1+ CD34+. Expression of CD34, also called hematopoietic stem cell (HSC) antigen, suggested these were blood-borne cells. The potential hematopoietic origin led the authors to explore the possibility that fibrocytes might arise from hematopoietic progenitors by utilizing a sex-mismatched BM chimeric mouse model. However, using the same wound chamber assay, Col1+ CD34+ cells of host origin only were detected; no Col1+ CD34+ donor BM-derived cells were observed. The authors interpreted these results as “*these data suggest that blood-borne fibrocytes do not originate from radiosensitive, hematopoietic stem cells but arise instead from either a radioresistant, bone marrow progenitor cell or from other tissue sources*”. Unfortunately, the authors did not determine whether fibrocytes could be derived from donor-derived PBMCs in chimeric mice. This would have shown that fibrocytes in the wound chamber and those isolated from the peripheral blood might be distinct populations. However, subsequent studies have identified donor BM-derived fibrocytes in other tissues including a HSC-derived fibrocytes in a dermal wound healing model suggesting the absence of donor BM-derived fibrocytes in the subcutaneous wound chamber may have been an assay specific finding (5).

### 2.1 Methods to Study Fibrocytes

The lack of a fibrocyte-specific marker has posed challenges to the investigation of this cell type *in vivo*. As a result, different strategies have been employed to study the contribution of fibrocytes to injury and disease models. These include: 1) immunolabeling, 2) labeling of circulating cells, 3) transgenic mice, and 4) transcriptional analysis which will be detailed in the following sections.

#### 2.1.1 Immunolabeling

**Figure 1:**
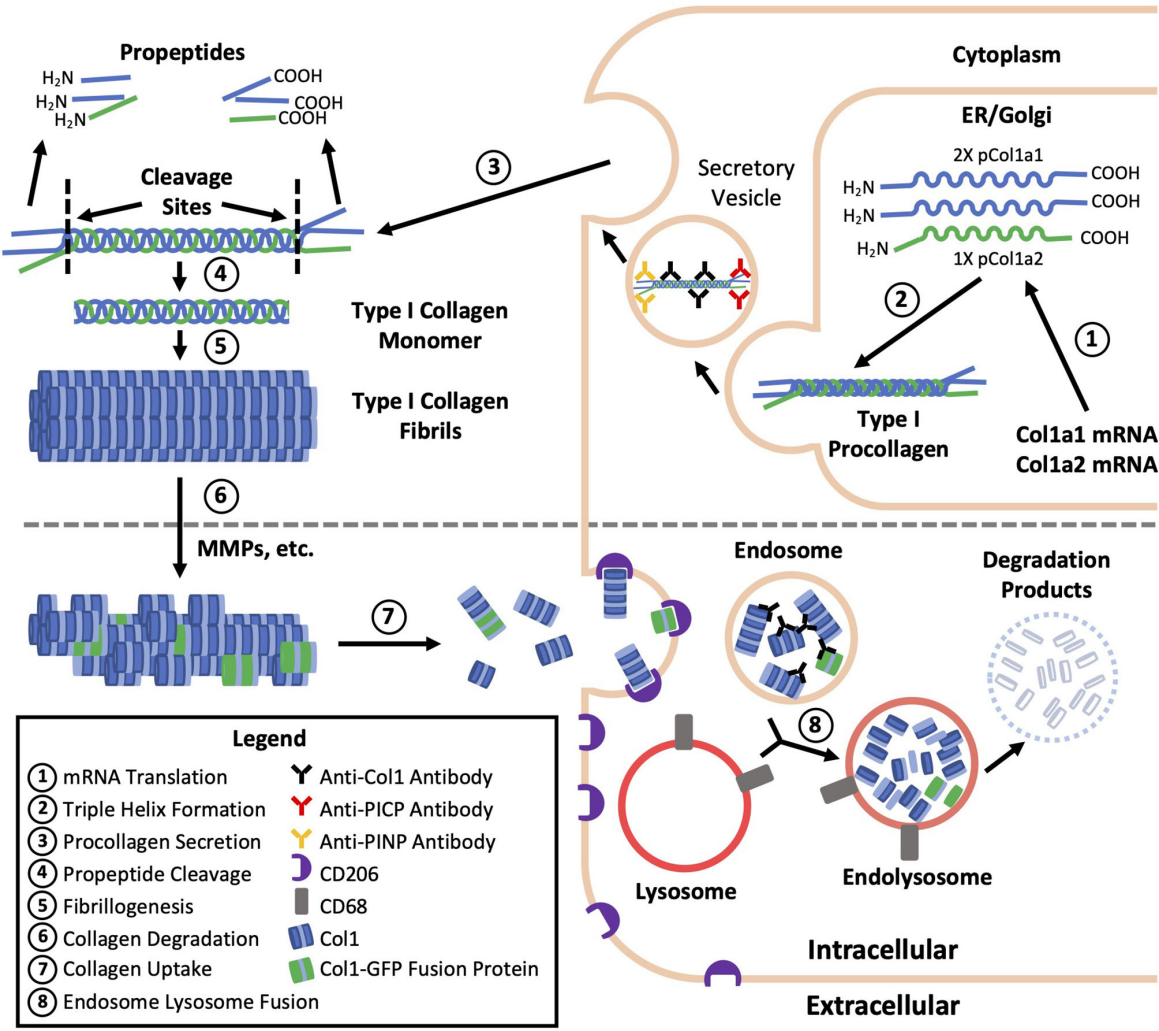
Type I collagen production, degradation, and uptake. The fundamental structural unit of type I collagen (Col1) is a triple helix comprised of two procollagen type I a1 (pCol1a1) chains and a single a2 (pCol1a2) chain. Each procollagen chain is flanked by C-terminal propeptide (PICP) and N-terminal propeptide (PINP) which are cleaved off in the extracellular space before a Col1 monomer can be incorporated into a fibril. Antibodies against Col1 are typically polyclonal, derived using purified Col1 or a synthetic peptide corresponding to a sequence within Col1a1 or Col1a2 as an antigen. Therefore, these antibodies against Col1 (black) do not recognize PICP or PINP which distinguish procollagen produced by a fibrocyte (top) from Col1 taken up by a macrophage (bottom) leading to the misidentification of some macrophages as fibrocytes. Use of transgenic mice expressing a collagen-GFP fusion protein (green, bottom) may also result in fibrocyte misidentification as the collagen-GFP fusion protein may be taken up by macrophages and present in endosomes prior to further degradation.

A common practice and limitation among studies of fibrocytes is the reliance on immunolabeling against Col1 as evidence of Col1 expression without validation of gene expression or protein production by a secondary method (**Table 1**). Validation is critical because immunolabeling only shows whether the protein is within the cell and does not distinguish whether this collagen has been produced by the cell or taken up from its environment (**Figure 1**) (6). Efficient uptake of collagen is believed to be a feature unique to mesenchymal cells with macrophages as the exception which can degrade collagen and uptake collagen fragments from their surroundings mediated by the M2 macrophage marker CD206 (1–3). To provide more context, the fundamental structural unit of Col1 is a triple helix comprised of two procollagen type I α1 (pCol1a1) chains and a single α2 (pCol1a2) chain. Each procollagen chain is flanked by procollagen type I C-terminal propeptide (PICP) and N-terminal propeptide (PINP) which are cleaved off in the extracellular space before a Col1 monomer can be incorporated into a fibril. Antibodies against Col1 are typically polyclonal, derived using purified Col1 or a synthetic peptide corresponding to a sequence within Col1a1 or Col1a2 as an antigen. Therefore, these antibodies do not recognize PICP or PINP which distinguish procollagen produced by a cell from Col1 that might have been taken up by the cell. While many studies report that positively immunolabeled cells “express” Col1, in this review cells that are positively immunolabeled will be identified by the target of immunolabeling followed by a “+’ symbol (e.g., Col1+).

**Table 1:** Anti-collagen antibodies.

Study Details				Antibody Information		
Ref.	Application(s)		Validation of Collagen Expression	Antibody (Immunogen)	Manufacturer	Catalog Number
(4)	FACS	Mouse Cells From Subcutaneous Wound Chamber	No	Anti-Collagen I	Chemicon International (Merck)	NS
	ICC	Human and Mouse Cultured PBMCs	No	Anti-Collagen I	Chemicon International (Merck)	NS
(6)	WB, IF, FC	Mouse Lung Cells and Cultured PBMCs	qPCR	Anti-Collagen I^A^	Rockland Immunochemicals	600-401-103
(7)	IF	Human Dermal Wound Biopsy Sections	qPCR	Anti-PINP (M-58)	Chemicon International (Merck)	MAB1912
(8)	IF	Mouse Vascular Graft Tissue Sections	NA	Anti-PICP	Sigma-Aldrich	ABT257
(9)	IF	Mouse Lung Tissue Sections	*In Situ* Hybridization	Anti-Procollagen I	Santa Cruz Biotechnology	NS
	FC	Human and Mouse PBMCs	No	FITC-Conjugated Anti-Collagen I	Chemicon International (Merck)	NS
(10)	IF, FC	Mouse Dermal Wound Biopsy Sections	qPCR	Anti-Collagen I^B^	Abcam	AB34710
	WB	Mouse Dermal Hematopoietic-Derived Cells		Anti-Collagen I^C^	Abcam	AB21286
(11)	ICC, FC	Mouse Lung Cells and Lung Fibroblast Cultures	No	Biotinylated-Anti-Collagen I^A^	Rockland Immunochemicals	600-406-103
(12)	IHC	Mouse Cardiac Infarct Tissue Sections	*Col1a2* Reporter Mice	Anti-Collagen I	Abcam	NS
(13)	IF	Mouse Dermal Granulation Tissue Sections	LCM-qPCR	Anti-Collagen I^D^	Abcam	AB19811
	IF	Mouse Dermal Granulation Tissue Sections	LCM-qPCR	Anti-Collagen I^B^	Abcam	AB34710
(14)	FC	Mouse PBMCs, Spleen Cells, and Kidney Cells	No	Biotinylated-Anti-Collagen I^A^	Rockland Immunochemicals	600-406-103
	IF	Mouse Kidney Tissue Sections	qPCR (Whole Tissue)	Anti-Collagen I	Abcam	NS
(15)	IF	Human Peripheral Blood Monocytes	No	FITC-Conjugated Anti-Collagen I (4F6)^E^	Southern Biotech	1441-02
	IHC	Mouse Heart Tissue Sections	No	Anti-Col1a1 (H-197)^F^	Santa Cruz Biotechnology	NS
(16)	IF, IHC, WB	Human and Mouse Kidney Tissue Sections	qPCR (Whole Tissue)	Anti-Collagen I	Southern Biotech	NS
	FC	Mouse Kidney Cells	No	FITC-Conjugated Anti-Collagen I	NS	NS
(17)	IF	Mouse Heart Tissue Sections	*Col1a1* Reporter Mice, qPCR (Whole Tissue)	Anti-Col1a	Abcam	NS
(18)	ICC, FC	Human Cultured PBMCs	NA	Anti-PINP (M-58)	Chemicon International (Merck)	MAB1912
(19)	ICC	Mouse Cultured Spleen Cells	No	Anti-Collagen I^A^	Rockland Immunochemicals	600-401-103
	FC	Mouse Cultured Spleen Cells	No	Anti-Collagen I^B^	Abcam	AB292
(20)	ICC, FC	Human Cultured PBMCs	No	Biotinylated-Anti-Collagen I^A^	Rockland Immunochemicals	600-406-103
(21)	FC	Human PBMCs	No	Anti-Collagen I^A^	Rockland Immunochemicals	600-401-103
(22)	ICC, FC	Human Cultured PBMCs	No	Anti-Collagen I	Chemicon International (Merck)	NS
	FC	Mouse BM Cells, Lung Cells, PBMCs	qPCR (Whole Tissue)	Anti-Collagen I	Chemicon International (Merck)	NS
(23)	IF	Human Heart Tissue Sections	No	Anti-Collagen I^A^	Rockland Immunochemicals	600-401-103
	IF	Mouse Heart Tissue Sections	NA	Anti-PICP (A-17)	Santa Cruz Biotechnology	SC25973
	FC	Mouse Heart Cells	IF for pCol1a1	Biotinylated-Anti-Collagen I^A^	Rockland Immunochemicals	600-401-103
(24)	ICC, IF	Mouse Cultured BM Cells, Mouse BM Tissue Sections	No	Anti-Collagen I^G^	Abcam	AB6308
	ICC, IF	Mouse Cultured BM Cells, Mouse BM Tissue Sections	No	Anti-Collagen I^B^	Abcam	AB34710
	FC	Mouse BM Cells	No	Biotinylated-Anti-Collagen I^A^	Rockland Immunochemicals	R1038B
(25)	IHC, FC	Mouse BM Tissue Sections and BM Cells	qPCR	Biotinylated-Anti-Collagen I^A^	Rockland Immunochemicals	600-406-103

NA, not applicable; NS, not specified; FC, flow cytometry; FACS, flow assisted cell sorting; ICC, immunocytochemistry; IHC, immunohistochemistry; IF, immunofluorescence; LCM, laser capture microdissection; WB, western blot; PICP, procollagen type I C-terminal propeptide; PINP, procollagen type I N-terminal propeptide.

A - Collagen type I from human and bovine placenta.

B - Full length native protein (purified) corresponding to human collagen I aa 1-1464. Collagen type I from human and bovine placenta.

C - Collagen type I extracted and purified from mouse skin.

D - Full length native human protein (purified).

E - Native human type I collagen.

F - Epitope corresponding to aa 1021-1217 of human Col1a1.

G - Collagen type I from bovine skin.

Assuming collagen propeptides cleaved off in the extracellular space are not taken up by hematopoietic-derived cells, these would be better markers to identify Col1 production (26, 27). Positive immunolabeling for PINP and CD45 has been used to identify fibrocytes in punch biopsies taken from a human partial-thickness dermal wounds caused by laser injury (7). One week after injury, in the upper dermis 15% of CD45+ cells were PINP+, accounting for 25.8% of total PINP+ cells. Three weeks following wounding, there were fewer CD45+ cells and PINP immunolabeling of CD45+ cells was not detected indicating a transient presence of CD45+ PINP+ cells in this model. Expression of pCol1a1 was confirmed by laser capture microdissection and immunomagnetic selection of CD45+ cells with mRNA expression measured by quantitative polymerase chain reaction (qPCR).

Unlike dermal wounds that resolve relatively quickly, implanted biomaterials trigger a foreign body response and prolonged inflammation. In a murine tissue engineered vascular graft abdominal inferior vena cava interposition model, fibrocytes were identified by immunolabeling for the mouse macrophage marker F4/80 and PICP (8). Three days after implanting bioresorbable polyester scaffolds, ~30% of F4/80+ cells were PICP+ and this increased to more than 80% of F4/80+ cells at day 14. The persistence of fibrocytes throughout longer periods of implantation and following complete scaffold degradation was not determined, so it is unclear whether these cells persist at the site of chronic inflammation or, like fibrocytes in the aforementioned resolving dermal injury model, have a transient presence.

*In situ* hybridization is a technique that can be used to identify gene expression within a histological section, overcoming the limitation of Col1 immunolabeling. By this method, a fluorophore labeled single-stranded RNA probe will localize to mRNA containing a complementary sequence. In biopsies of bronchial mucosa from patents with allergic asthma, to identify fibrocytes *in situ* hybridization for Col1a1 was performed complementary to immunolabeling for CD34 (9). The rationale for identifying fibrocytes based on Col1a1 and CD34 expression relied on cited literature asserting that fibrocytes are the only cell type to express these two markers, which has since proven to be inaccurate. CD34 and Col1a1 are also expressed by interstitial fibroblasts, mesenchymal stem cells (MSCs), and adventitial smooth muscle progenitors, though acquired expression of CD45 has not been reported on these cells (28–31). This study did not evaluate whether these *Col1a1* expressing CD34+ cells also expressed any hematopoietic markers.

#### 2.1.2 Labeling Circulating Cells

Labeling circulating cells has been used to demonstrate that fibrocytes contribute to intimal hyperplasia in an ovine carotid artery graft model using a gelatin-sealed Dacron graft (32). To label circulating cells, one day prior surgery 10% of the animal’s blood was harvested and leukocytes were isolated by density gradient centrifugation. Leukocytes were labeled with the fluorescent marker carboxyfluorescein diacetate succinimidyl ester (CSFE) and immediately reinjected. Animals were then sacrificed at 1, 2, and 4 weeks and grafts were analyzed by histology. Data to support the contribution of fibrocytes to intimal hyperplasia were presented as two pairs of images, one each at 2- and 4-weeks post implantation. Each pair consisted of one brightfield image of an immunohistochemical stain for alpha smooth muscle actin (αSMA), using Vector Blue as the chromogen, and the same section was imaged by fluorescence microscopy for CSFE. These images appear to show a considerable presence by CSFE labeled circulating leukocytes in the neointima that are absent in animals without CSFE labeled cells. In addition, greater than 50% of CSFE labeled cells expressed αSMA at both time points. While αSMA expression could be interpreted to imply myofibroblastic differentiation of a fibroblast-like (i.e., matrix producing) precursor, assessment of collagen production by CSFE labeled cells was not confirmed in this study. Therefore, it is possible for αSMA to have been expressed by these cells in the absence of collagen production and thus these cells were not rigorously identified as fibrocytes. A concern raised by the data presented is that the fractional area of CSFE labeling in the images seems far greater than would be expected based on labeling of 10% of circulating cells with CSFE unless the process of isolation and labeling were to greatly increase the propensity for these cells to preferentially home to the neointima compared to non-manipulated leukocytes. In addition, in the pair of 2-week images the overlap of αSMA and CSFE staining appears near one-to-one and intensities of labeling are consistent. It may have been overlooked that Vector Blue has broad excitation and emission spectra with peaks ~500 nm and 680 nm that overlap with the corresponding excitation and emission peaks for CSFE at 492 nm and 597 nm, respectively (33). The 4-week images and images from the control animal without CSFE labeled cells do not show the same consistency between Vector Blue and CSFE labeling, but the positively stained cells display a relatively round morphology, not the typical spindle-shaped morphology that would be expected for αSMA+ myofibroblasts and fibroblasts or that is present in the 2-week image.

These references to cell morphology within a histological section will be the first of many and the rationale for including this information will be provided here. During fibrocyte generation in culture from PBMCs, cell morphology provides some indication as to the state of differentiation since PBMCs are round at the time of plating and become elongated after adhesion and fibrocyte differentiation. Interpreting morphology from a histological section is less straightforward than for cells on a surface. A histological section is a cross-section of the 3-dimensional geometry present *in vivo*. Therefore, a cell may have a non-round morphology, but appear round due to the plane of sectioning. However, the converse is not true; a cell that appears to have a non-round morphology is not round. For a hematopoietic-derived cell, an elongated or spindle-shaped morphology alone is not sufficient evidence of the state of fibrocyte differentiation as a non-spherical morphology may reflect the local environment or result from an active behavior such as migration through a dense matrix. Therefore, while observations of cell morphology are included, morphology is only intended to be complementary to less subjective evidence of cell phenotype.

Tissue infiltration of labeled fibrocytes was characterized in a mouse model of allergic asthma induced by ovalbumin (9). Col1+ CD34+ fibrocytes were isolated from peripheral blood by density gradient centrifugation, enriched by immunomagnetic depletion, labeled with the red fluorescent membrane dye PKH-26, then intravenously transfused into recipient mice. Within 4 hours, PKH-26 positive cells could no longer be detected in the peripheral blood. 24 hours after transplantation, flow cytometry performed on single cell suspensions from these lungs showed slightly decreased immunofluorescence intensity for CD34 and increased immunofluorescence intensities for Col1 and αSMA on transplanted cells. Few labeled cells were observed in the lungs of PBS-treated control mice compared to the lungs of ovalbumin treated mice suggesting lung infiltration was a result of homing and not entrapment.

#### 2.1.3 Transgenic Mice

Transgenic mice enable cell labeling and lineage-tracing studies by incorporating foreign DNA into the genome such that transcription of a particular gene results in transcription of the foreign DNA. Location of insertion may be in genes that are globally expressed in all cells or a subset of cells in which gene expression is restricted. Common proteins encoded by the foreign DNA include fluorescent proteins or enzymes that facilitate colorimetric reactions for labeling. Transgenes may also include recombinases that enact genetic alterations and different transgenic mice may be cross-bred so that their progeny possesses multiple transgenes. A commonly used method to perform lineage tracing is the conditional Cre-lox model in which Cre recombinase is expressed under the control of a promotor for a cell type-specific gene and a second transgene containing a reporter is preceded by a stop sequence flanked by two LoxP recombination sites, also referred to as floxed (fl). An example is Cre recombinase expressed under the promotor for myeloid-specific lysozyme M (*LyzM-Cre*) using reporter green fluorescent protein (GFP) incorporated into the Rosa26 Cre reporter allele (*R26R-GFP*). Any cell expressing LyzM will also express Cre recombinase which will then excise the stop codon in the R26R Cre reporter allele (R26R to R26) allowing for GFP to be permanently expressed in that cell and its progeny. A disadvantage of conditional models is that if the cell-specific gene is expressed by a non-target cell type, as may occur during development or as a result of cell plasticity, the non-target cell and its progeny will also be permanently labeled with the reporter. For example, during wound healing and in cancer monocytes may express the endothelial marker Tie2 (34, 35).

A variation of lineage tracing is an inducible mouse model and one strategy by which this is done is by modifying the Cre transgene by fusing it to mutant estrogen ligand-binding domain (e.g., CreERT2). Only when a transgenic mouse expressing CreERT2 is treated with the estrogen mimetic tamoxifen will Cre translocate to the nucleus and induce recombination of the floxed sequence (36). Tamoxifen-dependent Cre recombinase allows for labeling to be performed at a specific time period during development or in an adult animal prior to a procedure, thus restricting recombination to a more highly-defined population of cells. A disadvantage of inducible Cre recombinase is “leakiness”, or Cre activity without tamoxifen administration. This may be addressed by including appropriate controls in the experimental design. Proper controls are also important as tamoxifen alone might interfere with certain models, as it is has been observed to cause cardiac fibrosis and DNA damage, and Cre activation has been linked to toxicity (37–41).

Multi-transgenic mouse models increase cell specificity, however trade-offs include a lower efficiency of labeling and more complex breeding schemes (42). Complementary strategies used in combination with transgenic mice include adoptive cell transfer, tissue transplant, and parabiosis, allowing cells from the donor mouse to be identified within the recipient or distinguishing cells between parabionts. In addition to colorimetric or fluorescent reporters, donor and host cells may be distinguished by sex-mismatching or using alleles encoding different isoforms for a particular gene (e.g., *CD45.1*/*CD45.2* and *CD90.1*/*CD90.2*). Many of the BM chimeric (**Table 2**) and other transgenic mouse models (**Table 3**) described in this review contain multiple transgenes. These will be listed in sequence separated by “x” to denote the cross-breeding that is typically done to generate multi-transgenic mice.

**Table 2:** BM chimera models.

Tissue	BM Donor	BM Recipient	Ref.
Skin	BALB/c (Male)	BALB/c (Female)	(4)
	*CAG-EGFP* (JAX #006567)	WT	(13)
		*CD90.1* (JAX #000406)	
	*Col1a1-GFPsapph* (43)	WT	(44)
	*Col1a2-EGFP* (45)	WT	(46)
	*Col1a2-Luciferase* (47)	WT	
	*SM22-Cre* x *R26R-LacZ* (JAX #004746, JAX #003474)	WT	(5)
Heart	*CAG-EGFP* (JAX #003291)	WT	(48)
	*CAG-EGFP* (49)	WT	(12)
	*Col1a1-GFP* (50)	WT	(51)
	*Col1a2-LacZ* (47)	WT	(12)
	*Col1a2-Luciferase* (47)	WT	
Kidney	*αSMA-RFP* (JAX #031159)	WT	(52)
	WT	*αSMA-RFP* (JAX #031159)	
	*Col1a1-GFP* (50)	WT	(53)
	*Cd11b-DTR* (JAX #006000)	WT	(14)
Liver	*Col1a1-GFP* (50)	WT	(54)
Lung	*CAG-EGFP* (55)	WT	(11)
PBMCs	*αSMA-GFP* (56)	WT	(56)
Femoral and Carotid Arteries	*CAG-EGFP* (49)	WT	(57)
Kidney, Liver, Lung, Heart, BM, Muscle	*Gli1-CreERT2* x *R26R-tdTomato* (JAX #007913, JAX #007909)	*CD45.1* (JAX #002014)	(29)

WT, wild type (C57BL/6); JAX, Jackson Laboratories.

**Table 3:** Other transgenic mouse models.

Tissue	Transgenic Mice	Model Details	Ref.
Skin	*Col1a1-topaz-GFP* (JAX #017466)	*Ex Vivo* Wound Macrophages	(13)
	*Col1a1-GFPsapph* (43)	Parabiosis with WT Mouse	(44)
	*LysM-Cre* (JAX #004781)	Conditional Reporter	(13)
	*R26R-mTmG* (JAX #007576)		
	*Vav-Cre* (JAX #008610)	Conditional Reporter	(10)
	*R26R-mTmG* (JAX #007676)		
Heart	*Col1a2-CreERT2*	Inducible Reporter	(17)
	*R26R-YFP*		
	*Col1a2-GFPtpz* (58)	Spleen-Derived Monocytes Transplanted to Peri-Infarct Myocardium	
	*LysM-Cre* (JAX #004781)	Conditional Reporter	(15)
	*R26R-EYFP* (JAX #006148)		
	*Vav-Cre*	Conditional Reporter Crossed to Col1a1 Reporter	(51)
	*R26R-tdTomato*		
	*Col1a1-GFP* (50)		
Kidney	*LysM-Cre* (JAX #004781)	Conditional Reporter with Renal Allograft (BALB/c Kidney Donor)	(16)
	*R26R-tdTomato* (JAX #007914)		
	*R26-CreERT2* (JAX #008463)	Inducible Reporter and Parabiosis with CD45.1 Mouse (JAX #002014)	(59)
	*R26R-tdTomato* (JAX #007909)		
Lung	*Col1a1-GFP* (50)	*Col1a1* Reporter	(6)
	*Vav-Cre* (JAX #008610)	Conditional Knockout	
	*Col1a1* ^fl^ (60)		

WT, wild type (C57BL/6); JAX, Jackson Laboratories.

#### 2.1.4 BM Chimeric Mouse Models

Since fibrocytes are hematopoietic-derived cells, *in vivo* studies of fibrocytes often utilize chimeric mice, generated by lethal irradiation to deplete host BM progenitor cells followed by BM reconstitution from a genetically distinct donor. This allows for distinguishing host cells from donor BM-derived cells and validation of this model typically reveals >90% of BM and circulating cells are donor-derived. However, an often overlooked aspect of BM transplantation is that in addition to HSCs, BM also contains MSCs that may become entrapped in the capillary beds throughout the body including tissues in which fibrosis is often studied such as the lung, liver, kidney, spleen, heart, and muscle (61). BM-MSCs are ~50% larger than BM hematopoietic cells and since BM transplantation is typically performed by intravenous infusion the pulmonary capillary bed will often be encountered first (62–64). Selective entrapment in the lungs imposes a limitation to the therapeutic use of intravenously delivered MSCs in other organs and limits BM engraftment (29, 65). The significance of BM-MSCs engrafting within organs is that MSCs are predisposed toward a fibrogenic (i.e., matrix producing) myofibroblast lineage and contribute to fibrosis in many organs (29). Under normal conditions and following organ injury, BM-MSCs do not circulate and should not contribute to fibrosis outside of the BM (29, 66–68). For fibrocytes, stimuli in the target tissue can result in the loss of monocyte and macrophage surface marker expression during fibrocyte differentiation making it difficult to distinguish hematopoietic- and mesenchymal-derived cells (10). Thus, when using chimeric mice generated by BM transplantation to study organ fibrosis, entrapment of MSCs in organs may inflate the perceived contribution of hematopoietic BM-derived cells in organ fibrosis following injury or disease. As an example, Gli1 is expressed on perivascular MSCs including BM-MSCs. Mice with the *CD45.1* isoform were lethally irradiated and reconstituted with whole BM from *Gli1-CreERT2* x *R26R-tdTomato* x *CD45.2* mice that were induced with tamoxifen prior to sacrifice and BM harvest (29). Following transplantation, only a small number of *Gli1*-derived donor cells were found in the BM and kidneys, but many had become entrapped in the lungs and could still be found 6 weeks after transplantation with some expressing the myofibroblast marker αSMA. A strategy that can alleviate potential concerns associated with the engraftment of BM-MSCs throughout the body is to perform sorting to enrich the CD45+ or HSC fractions of BM prior to transplantation. This has been accomplished successfully with mice surviving and reaching high levels of peripheral blood chimerism with greater than 90% of circulating cells being donor-derived (5).

#### 2.1.5 Ubiquitous Donor BM Cell Labeling

Bleomycin-induced lung fibrosis was performed in chimeric mice generated by transplanting BM from GFP donors into lethally irradiated wild type (WT) recipients. Flow cytometry performed on disaggregated cells from lung tissue found 27.5% of cells expressed GFP and were Col1+, representing 80% of Col1+ cells (11). When GFP+ cells isolated from lung tissue were cultured, these cells adopted a spindle-shaped, fibroblast morphology, however none expressed the myofibroblast marker αSMA and they were resistant to TGF-β1-induced myofibroblast differentiation. In a myocardial infarction (MI) model of heart fibrosis, coronary artery ligation was performed on chimeric mice generated by transplanting BM from enhanced GFP (EGFP) mice into lethally irradiated WT recipients (12). At day 7 post-MI, BM-derived αSMA+ cells peaked, averaging 21% of BM-derived cells and 24% of αSMA+ cells. In another study using a similar chimeric mouse model, following coronary artery ligation 57% of myofibroblasts (vimentin+ αSMA+) were BM-derived (48). In addition to the difference in how fibrocyte-derived myofibroblasts were identified, a noticeable difference between the two studies that might account for the difference in the percentage of BM-derived myofibroblasts is that the latter study transfused 3-5-fold more donor BM cells into recipient mice. In a splinted dermal excisional wound healing model, BM-derived cells were tracked using chimeric mice generated by transplanting BM from EGFP donors into lethally irradiated WT recipients (13). At day 5, multicolor immunofluorescence identified EGFP+ F4/80+ Col1+cells within the wound.

#### 2.1.6 Lineage Tracing

Vav-1 is a pan-hematopoietic marker expressed almost exclusively in hematopoietic cells (10, 69). Vav-1 has advantages over CD45, regarded as the most specific marker for hematopoietic cells, because CD45 is expressed during later stages of hematopoietic progenitor cell development and incompletely identifies hematopoietic-derived cells (70). Transgenic *Vav-Cre* x *R26R-mTmG* mice have been used to identify fibrocytes in a dermal excisional wound model (10). In *mTmG* mice, cells express membrane-targeted tandem dimer Tomato (tdTomato), a red fluorescent protein (RFP), prior to Cre-mediated excision and membrane-targeted GFP after excision. In *Vav-Cre x R26R-mTmG* mice, nearly all peripheral blood cells were GFP+ and circulating RFP+ cells were not detected. Single-cell qPCR performed on cells isolated from the wound identified expression of *Col1a1*, *Col1a2*, and *Col3a1* by monocytes and macrophages that peaked at day 7 post wounding. Of these cells, 5% had low expression of CD45 and 60% had low expression of CD11b showing that use of these markers would have under identified fibrocytes. Another study employing a lung fibrosis model selectively knocked out production of Col1 in hematopoietic-derived cells by using *Vav-Cre* x *Col1a1^fl^* mice (6). Fibrocytes with confirmed deletion of the *Col1a1* gene still immunolabeled for Col1, lending further support that positive immunolabeling for Col1 is not reliable strategy for identifying fibrocytes as it does not discriminate Col1 expression from uptake of Col1 from the surrounding environment. An important detail to consider when interpreting these findings is that the antibody used in this study was polyclonal, generated using Col1 as the antigen, and may exhibit reactivity with pCol1a2. Therefore, positive immunolabeling by this Col1 antibody may not definitively identify uptake of collagen in cells lacking the ability to express *Col1a1* but retaining the ability to express *Col1a2*.

CD11b is expressed on fibrocytes, monocytes, granulocytes, and NK cells; therefore, CD11b is more selective for fibrocytes than pan-hematopoietic labeling based on Vav-1 or CD45 expression. However, while CD11b is expressed on a majority of these cells its expression is not ubiquitous so this is not regarded as an efficient marker. In a unilateral ureteral obstruction (UUO) model of kidney fibrosis, ablation of CD11b expressing cells was performed using CD11b-DTR mice (14). In these mice, expression of the diphtheria toxin receptor (DTR) is under the control of the CD11b promotor and administration of diphtheria toxin (DT) results in depletion of CD11b+ cells. Chimeric mice were generated by transplanting BM from *CD11b-DTR* donors into lethally irradiated WT recipients. These chimeras allow for selective depletion of *CD11b* expressing BM-derived cells and not other BM-derived cells or host cells that express *CD11b*. A single injection of DT reduced peripheral blood monocytes by ~50% and subsequent injections almost completely depleted monocytes in the blood and obstructed kidney. Following monocyte depletion, in the spleen Col1+ fibrocytes were reduced by nearly two-thirds and in the obstructed kidney Col1+ fibrocytes were depleted or infiltration had been impaired.

Lysozyme M (LysM) is expressed on monocytes, macrophages, and granulocytes. Assuming granulocytes maintain their unique surface marker expression, they can be distinguished from monocytes and macrophages by immunolabeling, such as for expression of myeloperoxidase by neutrophils (13). Coronary artery ligation-induced MI was performed in *LysM-Cre* x *R26R-EYFP* mice that use enhanced yellow fluorescent protein (EYFP) as the reporter and remodeling of the infarct was tracked for 6 weeks (15). *LysM*-derived cells in the infarct region possessed a round morphology at 1 week and some possessed an elongated morphology at 6 weeks. *LysM*-derived Col1+ cells were observed at 1 week and Col1 immunolabeling appeared relatively more intense at 6 weeks. A separate study used similar LysM lineage reporter mice in a renal allograft model of chronic rejection in which BALB/c kidneys were transplanted into *LysM-Cre* x *R26R-tdTomato* recipient mice (16). At the 28 day endpoint, in the donor kidney most F4/80+ macrophages were tdTomato+ host-derived cells and many of these were Col1+ αSMA+. The translational relevance of these findings was demonstrated in biopsies performed on human renal allografts. Immediately following transplantation, no cells co-expressed the macrophage marker CD68 and αSMA. In acute renal allograft rejection, there was a greater number of CD68+ macrophages and a few expressed αSMA. However, in chronic renal allograft rejection there were many αSMA+ cells in regions with severe interstitial fibrosis, half expressed CD68, and some were Col1+. A caveat with these findings is that the choice to identify fibrocytes by CD68 and αSMA expression might be misleading as during wound healing SMCs that normally express αSMA may also express macrophage markers including CD68 and Mac-3 (71, 72). A different study used *LysM-Cre* x *R26R-mTmG* mice to characterize fibrocytes in a murine splinted excisional wound model (13). *LysM*-derived cells isolated from the wound edge by laser capture microdissection expressed *Col1a1* at days 3 and 7 post wounding as determined by qPCR.

Since by definition fibrocytes must produce collagen, a complementary strategy to those previously mentioned for identifying fibrocytes is to use a reporter of collagen expression (e.g., *Col1a1-GFP*) and identify labeled cells that also express a hematopoietic marker (12, 46). This approach circumvents concerns regarding the use of Col1 immunolabeling to identify fibrocytes. In a cardiac fibrosis model, coronary artery ligation was performed on chimeric mice generated by transplanting BM from *Col1a1-GFP* donors into lethally irradiated WT recipients (51). In response to coronary artery ligation or placement of a suture in sham mice, GFP+ cells that co-expressed CD45 and possessed a round morphology were observed at the pericardial surface, but not within the infarcted myocardium. The hematopoietic origin of these cells was confirmed following coronary artery ligation in *Vav-Cre* x *R26R-tdTomato* x *Col1a1-GFP* mice as these GFP+ cells were also tdTomato+. In a UUO kidney fibrosis model, chimeric mice were generated by transplanting BM from *Col1a1-GFP* donors into lethally irradiated WT recipients (53). A small number of GFP+ CD34+ CD45+ cells were detected exclusively in perivascular areas and were typically found surrounding venules in fibrotic kidneys, but BM-derived cells were not found in normal kidneys or the contralateral unobstructed kidney. However, GFP+ cells accounted for only 1 out of every 1000 *Col1a1* expressing cells. In a bile-duct ligation induced liver injury model, chimeric mice were generated by transplanting BM from *Col1a1-GFP* donors into lethally irradiated WT recipients. BM-derived cells localized to the site of injury and represented ~5-10% of *Col1a1* expressing cells (54). In a splinted dermal excisional wound healing model, *Col1a1* expression by wound macrophages was discovered to be induced by keratinocyte-derived miR-21 (13). Macrophages isolated from dermal wounds of *Col1a1-*(*topaz-GFP*) mice at day 3 were treated with a miR-21 mimic and showed several-fold greater expression of GFP compared to treatment with non-specific miR. Keratinocyte stimulated collagen production demonstrates tissue-specific signaling pathways may enable certain tissues an advantage over other tissues at promoting collagen production by hematopoietic cells.

In a recent study, to determine whether monocytes and macrophages directly participate in scar formation following MI, coronary artery ligation was performed on *Col1a2-CreERT2* x *R26R-YFP* mice at postnatal day 7 (P7) and Cre was activated at day 5 post-MI (17). Multicolor immunofluorescence identified CD68+ macrophages that were YFP+, however analysis of the single color image for the YFP channel on the representative image showed that YFP expression in CD68+ cells was weak relative to YFP+ CD68- cells and YFP signal was detected in only a fraction of the cell area immunofluorescent for CD68. In this transgenic mouse model, Cre-mediated recombination should result in stable expression of YFP. Weak YFP expression in CD68+ cells relative to CD68- cells raises the question as to whether the CD68+ cells are truly expressing YFP or whether the YFP fluorescence signal in CD68+ cells might have other explanations that might include autofluorescence, which is a feature of heart tissue, a YFP+ CD68- cell and a YFP- CD68+ cell slightly out of plane with each other, or phagocytosis of apoptotic YFP+ cells by CD68+ cells.

In separate experiments within the same study, to determine whether monocyte-derived cells produce collagen that contributes to scar formation post coronary artery ligation, donor splenic monocytes from *Col1a2-GFPtpz* mice that express a collagen-GFP fusion protein were enriched by immunomagnetic negative selection and injected into the peri-infarct myocardium of adult WT mice at the time of coronary artery ligation. At day 7 post-MI, within the scar region GFP signal colocalized almost exclusively with CD68+ cells, though not all CD68+ cells colocalized with GFP. The latter reflects CD68+ cells of donor origin that were not producing collagen and CD68+ cells of host origin regardless of collagen expression. Given that macrophages can take up collagen, the GFP+ CD68+ population may include non-collagen producing cells of donor or host origin that had recently internalized GFP labeled collagen. At day 21 post-MI, Col1 immunolabeling was highly coincident with the GFP signal. To state this differently, it appeared as though nearly all Col1 fibers contained the collagen-GFP fusion protein. In the absence of donor monocyte injection 75% of *Col1a1* expressing cells in the heart following coronary artery ligation were identified as fibroblasts compared to 5.5% of *Col1a1* expressing cells identified as macrophages. While the intramyocardial injection of splenic monocytes would be expected to result in a localized region containing an artificially high *Col1a2-GFPtpz* donor monocyte to host WT fibroblast ratio, the apparent lack Col1 fibers not containing collagen-GFP remains a bit surprising. A challenge when performing immunofluorescence on collagen containing tissue is autofluorescence of collagen. While the authors attempted to address this potential issue by immunolabeling GFP and Col1, the representative images presented call into question whether these efforts were sufficient to avoid this phenomenon. An unaddressed concern that could impact the findings of this study as well as others using transgenic mice expressing a collagen-GFP fusion protein is the potential immunogenicity of GFP. GFP is a foreign protein and the immune response to GFP may enhance macrophage uptake of collagen fibers containing the collagen-GFP fusion protein (73).

#### 2.1.7 Parabiosis

Parabiosis is the surgical joining of two organisms which results in a shared circulatory system. An advantage of parabiosis models over chimeric mice is that one need not be concerned with potential engraftment of MSCs in tissues throughout the body or trauma as a result of irradiation. A modest disadvantage is that chimerism results in a 50:50 representation of cells from each parabiont within the blood. To look at the contribution of circulating fibrocyte-derived myofibroblasts in UUO model of kidney fibrosis, one study used a parabiosis model in which the parabiont to later undergo kidney fibrosis possessed the *CD45.1* isoform and the other parabiont was *R26-CreERT2* x *R26R-tdTomato* x *CD45.2* (59). In the latter parabiont, CreERT2 is constitutively expressed in all cells and tamoxifen administration should result in tdTomato expression. Therefore, cells from this parabiont should be distinguishable by both CD45.2 and tdTomato expression. Ten days following UUO surgery, more than half of CD45+ cells in the UUO kidneys were derived from CD45.2 parabiont and tdTomato+ cells accounted for 10% of αSMA+ cells. A different study sought to determine if BM-derived fibrocytes contribute to excisional dermal wound healing by using a parabiosis model in which the parabiont to receive the dermal wound was WT and the other parabiont possessed the *Col1a1-GFP* transgene (44). Only a few GFP+ cells could be detected in the wound of the non-transgenic parabiont. GFP+ cells possessed a wide range in morphology from round to spindle-shaped and some expressed Mac-3, but an acknowledged caveat is that the expression of the visual *Col1a1-GFP* transgene that was used for cell identification in this study may not always correlate with endogenous *Col1a1* gene expression within myeloid cells.

#### 2.1.8 Transcriptional Analysis

In studies of fibrocytes, transcriptional analyses have traditionally relied on qPCR which quantifies the amount of known sequences of RNA relative to a reference gene. Compared to qPCR performed on a mixed population of cells isolated from whole tissue, more insightful data is generated when qPCR is performed on purified populations or even single cells. In contrast, next generation sequencing methods such as RNA-Seq enable quantification of gene expression without requiring prior knowledge of sequence information thereby allowing for a broader, unbiased assessment of gene expression. scRNA-Seq is particularly revealing as it aids in cell phenotyping and enables the assessment of heterogeneity within cell subpopulations. As costs come down and accessibility to next generation sequencing methods increases, single-cell transcriptional analyses are increasingly being employed that promise to accelerate our collective understanding of fibrocytes.

In a coronary artery ligation model performed in *Col1a1-GFP* mice, 7 days following MI bulk RNA-Seq was performed on macrophages isolated from hearts and enriched by flow assisted cell sorting (17). Compared to mice in which ligation was performed at postnatal day 1 (P1), when ligation was performed at P7, in macrophages there was upregulation of *Col1a1*, *Col1a2*, and *Col3a1* as well as additional ECM genes including *Col8a1*, *Col5a2*, *Col6a2*, *Fbn1*, *Postn*, and *Bgn*. Gene expression was not measured in a reference cell type, such as a cardiac fibroblast, so no comparison can be made as to gauge relative collagen production by these cardiac macrophages. In a separate study, scRNA-Seq was performed on cells from a mouse non-splinted excisional wound model 12 days post wounding (5). Myeloid-derived cells represented 15% of all cells within the wound and myofibroblasts expressing *LysM*, *Col12a1*, and the contractile markers *αSMA* and *SM22* represented ~11% of all wound fibroblasts. Pseudotime analysis showed the conversion of *LysM* expressing myeloid cells into fibroblasts. Using *SM22-Cre* x *R26R-tdTomato* mice, single-cell western blot confirmed that 6% of SM22 expressing cells were LYZ+. Immunostaining further confirmed the presence of LYZ+ αSMA+ cells within wounds. scRNA-Seq performed on cells isolated from 15 and 21 day wounds showed that a population of fibrocyte-derived myofibroblasts persisted within wounds. The possibility that cell fusion of BM-derived cells and fibroblasts could account for the apparent generation of BM-derived fibrocytes was ruled out in chimeric mice generated by transplanting GFP+ HSCs into lethally irradiated RFP+ recipients. While cells expressing both GFP and RFP were observed, their presence was rare within wounds. However, this experiment did not address potential fibrocyte generation by fusion of a radioresistant, tissue resident macrophage with a fibroblast.

### 2.2 Collagen Production by Fibrocytes

Few studies have attempted to determine the amount of collagen produced by fibrocytes relative to overall collagen production during fibrosis. *In vitro*, cultured pulmonary fibroblasts expressed 13-fold greater *Col1a1* and 34-fold greater *Col3a1* mRNA on a per cell basis than PBMC-derived fibrocytes (2). Correspondingly, significantly greater collagenous protein was produced by cultured pulmonary fibroblasts than PBMC-derived fibrocytes. Human PBMC-derived fibrocytes from normal subjects and burn patients produced substantially less hydroxyproline *in vitro* than dermal fibroblasts and weakly expressed pCol1a1 on a per cell basis compared to cultured normal human dermal fibroblasts (18, 74). Regarding the relevance of *in vitro* experiments to collagen expression by fibrocytes *in vivo*, a different study compared the expression of Col1a1 mRNA from CD45+ cells isolated from a human dermal wound at 1 week to human fibrocytes differentiated from PBMCs for 10 days and found comparable expression (7).

*In vivo*, collagen production by fibrocytes has been investigated in dermal wound and fibrosis models in *Col1a2-luciferase* mice. Non-splinted dermal excisional wounds were performed on these transgenic mice as well as chimeric mice generated by transplanting BM from *Col1a2-luciferase* donors into lethally irradiated WT recipients (46). Luciferase activity was almost non-existent in chimeric mice compared to *Col1a2-luciferase* mice indicating that BM-derived cells do not directly contribute significant collagen production to healing dermal wounds. In a separate experiment, bleomycin-induced dermal fibrosis was performed on chimeric mice generated by transplanting BM from *Col1a2-EGFP* donors into lethally irradiated WT recipients. 21 days after induction, the fibrotic tissue contained spindle-shaped and round EGFP+ cells that represented ~5% of Col1a2 producing cells, though the relative amount of collagen produced by BM-derived cells compared to non-BM-derived cells was not determined.

In a splinted dermal excisional wound healing model, deletion of miR-21 in keratinocytes resulted in a decrease in Col1+ macrophages at the wound site, deficiency in wound granulation tissue collagen content, and decreased stiffness of the repaired skin (13). However, the relative number of fibrocytes to total collagen producing cells was not quantified nor was the effect of miR-21 on non-fibrocyte collagen producing cells. Therefore, it is unclear how significant collagen produced by macrophage-derived fibrocytes is to overall collagen produced within a healing dermal wound. In addition, splinting results in increased tension in the healing wound which may increase fibrosis (75). The effect of splinting on fibrocyte differentiation and collagen production by fibrocytes was not compared to non-splinted wounds, so it is not apparent how relevant collagen production by fibrocytes in splinted wounds is to the more physiological setting of dermal wound healing.

In a coronary artery ligation model performed on *Col1a1-GFP* mice, flow cytometry was used to characterize cells isolated from the heart at day 7 post MI (17). F4/80+ macrophages comprised 0.45% of total cells compared to 6.1% for fibroblasts, but 46.2% of F4/80+ cells were GFP+ compared to just 22.5% of fibroblasts. Overall, F4/80+ cells accounted for 6.8% of GFP+ cells. Following up on these observations, BM-derived macrophages from *Col1a2-GFPtpz* mice expressing a GFPtpz-collagen fusion protein were co-cultured with L929 fibroblasts. GFP+ collagen fibers could be seen in close proximity to CD68+ macrophages. The high resolution confocal images of a single macrophage revealed a punctate staining pattern for CD68 and GFP consistent with intracellular vesicles. If this cell was producing collagen, then the GFP signal should be associated with secretory vesicles. CD68, also known as lysosome associated membrane protein 4 (LAMP4), is often associated with the endosomal/lysosomal compartment. While nearly all CD68+ immunolabeling overlapped with GFP, there were regions of GFP signal absent for CD68 immunolabeling. This pattern of staining raises the question as to whether this GFP labeled collagen is being secreted and/or degraded by the single macrophage imaged. For this experiment, BM-derived macrophages were isolated by plating BM cells and selecting for adherent cells after 7 days of culture. This is similar to the procedure of isolating fibrocytes from the blood which involves density gradient centrifugation, plating of cells from the buffy coat to select for adherence, and culture for up to 14 days in serum containing medium to enrich for cells that have an elongated morphology (30, 76). Unlike PBMCs, BM cells contain fibroblasts which are also adherent cells and no characterization was reported to validate the purity of these BM-derived macrophages. Therefore, the concern raised by the details provided is that the BM-derived macrophages may have been contaminated with fibroblasts, collagen fibrils observed may have been produced by these contaminating fibroblasts, and the macrophage pictured may have been in the process of degrading and taking up this collagen, not producing it.

After observing that fibrocytes produced less collagen than dermal fibroblasts, as measured by a hydroxyproline assay, one study sought to characterize the indirect role of fibrocytes on collagen production by dermal fibroblasts (18). Cultured dermal fibroblasts were treated with conditioned medium from cultured fibrocytes derived from the peripheral blood. Fibrocyte conditioned medium from burn patients, but not from normal subjects, increased fibroblast proliferation and migration, αSMA expression, and contraction of collagen gels. These effects were determined to be caused by elevated TGF-β1 in conditioned medium from burn patient fibrocytes that could be counteracted by using a TGF-β1 neutralizing antibody.

The consensus appears to be that fibrocytes make up a small percentage of collagen producing cells and that on a per cell basis fibrocytes produce far less collagen than fibrogenic cells to which they have been compared. Not to dismiss the direct contribution of fibrocytes to fibrosis, but it seems that fibrocytes as well as monocytes and macrophages from which fibrocytes may be derived have a greater indirect role on fibrosis. These hematopoietic-derived cells produce growth factors and cytokines that stimulate recruitment, proliferation, matrix production, and myofibroblastic differentiation by traditional fibrogenic cells in addition to stimulating their own fibrocyte differentiation in an autocrine manner (77, 78). Systemic depletion of monocytes and macrophages significantly reduces tissue fibrosis, a finding that appears independent of fibrocyte activity (14, 79). Therefore, antifibrotic strategies that target monocytes and macrophages upstream of fibrocyte differentiation might be more effective, analogous to killing two birds with one stone.

### 2.3 Fibrocyte-Derived Myofibroblasts

To properly evaluate studies reporting fibrocyte-derived myofibroblasts, one must first understand the criteria for a cell to be identified as a myofibroblast. Tomasek et al. described a two-stage model of myofibroblast differentiation from fibroblasts (80). Growth factors released during the early stages of wound healing activate fibroblasts, stimulate their migration toward the site of injury, and induce the expression of the ED-A splice variant of fibronectin. As fibroblasts begin to reorganize the collagen matrix by exerting traction forces, they develop cytoplasmic beta- and gamma-actin containing stress fibers and upregulate expression of ED-A fibronectin. Subsequent exposure to TGF-β1 in the sustained presence of ED-A fibronectin and mechanical stress promotes the expression of αSMA, stress fiber development, expansion adhesion complexes, as well as increases the production of ECM proteins, features that collectively establish the myofibroblast phenotype. The presence of αSMA is significant because it enables cells to generate greater contractile force than non-αSMA expressing cells and is a feature that grants myofibroblasts the prefix “myo” to denote that these cells are “smooth-muscle-like” (80, 81).

Though the myofibroblast phenotype is defined by multiple distinctive features, αSMA is a commonly accepted marker for identifying fibroblast-derived myofibroblasts. Using αSMA expression to identify fibrocytes or myofibroblasts derived from hematopoietic cells is relatively more problematic because it presumes collagen expression and this is not a safe assumption; *in situ* hybridization performed on heart tissue in a coronary artery ligation model found that 30% of αSMA expressing cells did not express *Col1a1 (* 82). In the absence of demonstrated collagen expression, it would arguably be more accurate to refer to αSMA+ hematopoietic-derived cells by the protologism myohematopoietic cells, or perhaps more specifically as myomyeloid cells. However, so as to not introduce potential confusion, αSMA+ hematopoietic-derived cells will be referred to as αSMA+ or as myofibroblasts in keeping with the nomenclature from the cited source.

Fibrocyte-derived myofibroblasts have been generated and studied *in vitro*. Freshly isolated human PBMCs did not express αSMA, as determined by qPCR, but αSMA mRNA was detected after 10 days in culture (20). Expression increased 4-fold and kinetics of expression could be increased when the culture medium was supplemented with TGF-β1 (20, 22). Another study reported that in cultures of Col1+ CD34+ cells isolated from the peripheral blood, after 6 days in serum free medium 20% expressed αSMA, but more than 80% expressed αSMA in the presence of TGF-β1 (9). The contractile ability of αSMA+ fibrocytes has been assessed by suspending cells within a collagen gel (20). After 72 hours, PBMCs contracted gels by ~5% compared to ~20% for cultured fibrocytes, ~30% for fibrocytes cultured in the presence of TGF-β1, and 40% for dermal fibroblasts. Unlike fibrocyte collagen production, which has been measured to be far below that of dermal fibroblasts, based on this one study collagen gel contraction appears relatively similar between fibrocytes and fibroblasts. In future studies of fibrocyte-derived myofibroblasts, perhaps greater emphasis should be placed on their contribution to contraction-mediated ECM remodeling than collagen production.

*In vivo*, the presence of BM-derived cells expressing αSMA appeared to be dependent on the tissue and/or the model used. Flow cytometry showed that in healthy humans a small minority of circulating Col1+ CD45+ fibrocytes were positive for αSMA with no significant difference observed in patients with interstitial pneumonia (21). In *αSMA-GFP* mice, 0.15% of circulating mononuclear cells were GFP+, although the average intensity of GFP was far lower than in aortic smooth muscle cells from the same transgenic mice indicating lower *α*SMA expression (56). A detail to consider when interpreting these findings is that the half-life of unmodified GFP in mammalian cells is reported to be ~26 hrs, so circulating cells may have stopped translating αSMA protein days ago (83). In a lung allergic asthma model induced by ovalbumin, 24 hours following intravenous administration of labeled fibrocytes αSMA expression had increased in labeled cells isolated from lung tissue (9). In the heart, following coronary artery ligation αSMA was detected in BM-derived cells and in *Col1a2*-derived BM cells, but not among *LysM*-derived BM cells (12, 15, 48). In kidney fibrosis studies, one study did not find αSMA expression on *Col1a1*-derived BM cells, a second study using a parabiosis model found that 10% of αSMA+ cells could be identified as originating from the parabiont, and a third study found that 35% of αSMA+ cells were BM-derived, but did not confirm collagen production by these cells (52, 53, 59). The contribution of BM-derived cells to neointimal and medial cells in vascular injury models was investigated in chimeric mice generated by transplanting *CAG-GFP* BM into lethally irradiated WT recipients (57). In response to wire-induced injury, many BM-derived GFP+ cells in the intima and media expressed αSMA, but few expressed αSMA in response to cuff- or ligation-induced injury. In a bile-duct ligation induced liver injury model, chimeric mice were generated by transplanting BM from *Col1a1-GFP* donors into lethally irradiated WT recipients. BM-derived cells localized to the site of injury and represented ~5-10% of *Col1a1* expressing cells, but did not express αSMA (54). In a bleomycin-induced dermal fibrosis model performed on chimeric mice generated by transplanting BM from *Col1a2-EGFP* mice into lethally irradiated WT recipients, αSMA was expressed in a small number of the EGFP+ semi-round cells, but not in most of the spindle-shaped cells (46).

Conflicting findings regarding the presence of αSMA+ BM-derived cells have been reported in dermal wound and fibrosis models. In a splinted dermal excisional wound model performed in *Vav-Cre* x *R26R-mTmG* mice, 7 days following wounding many *Vav*-derived cells expressed αSMA, but nearly all cells possessed a round morphology (10). After 14 and 28 days, αSMA expression on hematopoietic-derived cells had diminished compared to day 7. Though collagen production in hematopoietic-derived cells expressing αSMA was not demonstrated *in vivo*, qPCR performed on primary cultures of BM-derived CD11b+ cells showed that TGF-β1 increased the expression of both Col1 and αSMA. In addition, single cell qPCR performed on monocytes and macrophages isolated from dermal wounds at day 7 and bulk qPCR performed on cultured CD11b+ cells showed the production of Col1 by these cells. In contrast to these findings, in a non-splinted dermal excisional wound model performed on chimeric mice generated by transplanting BM from *Col1a2-EGFP* donors into lethally irradiated WT recipients, the number of EGFP+ cells peaked at day 7, but no EGFP+ cells expressed αSMA (46). Similar findings were reported in a parabiosis model in which the parabiont to receive a non-splinted excisional dermal wound was WT and the other parabiont possessed the *Col1a1-GFP* transgene (44). Only a few GFP+ cells could be detected in the wound of the non-transgenic parabiont and all GFP+ cells were negative for αSMA. The presence of BM-derived αSMA+ cells in the splinted wounds but not non-splinted wounds may be explained by the increased mechanical tension present in the splinted wound, a factor recognized to promote αSMA expression and myofibroblast differentiation (75). Alternatively, if the difference in αSMA expression was simply due to the different transgenic mouse models, it is possible that αSMA expression was similar between splinted and non-splinted wounds, but that αSMA was not observed in BM-derived cells in the non-splinted wounds because it was expressed in the fraction of BM-derived cells not expressing *Col1a1* or *Col1a2*.

PDGFRβ is another marker that has been used to identify fibrocyte-derived myofibroblasts (59). In a UUO model of kidney fibrosis, circulating “hematopoietic-derived myofibroblasts” were identified as PDGFRβ+ CD45+ and non-hematopoietic tissue resident cells as PDGFRβ+ CD45-. The justification for using PDGFRβ to identify myofibroblasts was that this marker has been reported to be expressed on myofibroblasts and circulating fibrocytes. However, it was not addressed whether non-myofibroblasts present in the UUO kidney or circulation might also express PDGFRβ. Therefore, while PDGFRβ may have a high sensitivity for identifying myofibroblasts, its specificity, or the rate of false positives was not presented. Accordingly, scRNA-Seq performed on these two populations showed that gene expression of hematopoietic-derived, PDGFRβ+ CD45+ cells correlated strongly with human peripheral blood monocytes and the expression of matrix genes and αSMA was low compared to tissue resident-derived, PDGFRβ+ CD45- cells. While circulation-derived cells provided a small contribution to the population of myofibroblasts, they were found to express a relatively high amount of chemokines and interleukins indicating they may have a greater indirect role in regulating kidney fibrosis by secreting paracrine factors.

### 2.4 Potential Origins of Fibrocytes

In healthy animals, at the time of isolation ~1% of PBMCs were Col1+ CD34+ giving the appearance circulating fibrocytes were already differentiated or partially differentiated cells (9, 14, 84, 85). Col1+ CD45+ cells can also be found in the BM of healthy mice suggesting that perhaps fibrocytes differentiate prior to entering the circulation (24, 25). However, it remains to be determined whether fibrocytes might differentiate from a distinct fibrocyte precursor or from myeloid cells (e.g., monocytes) within the BM. Regarding the latter possibility, in the original study that identified fibrocytes, fibrocytes isolated from the wound chamber assay appeared distinct from monocytes because they lacked expression of CD14 and non-specific esterases. This should not be interpreted to exclude monocytes as a potential origin for fibrocytes. In fact, a 20-year follow-up article by the first author of the foundational study recognized that fibrocytes arise from circulating monocyte precursors (86). Also, a 2001 study found that *in vitro* fibrocytes could only be differentiated from the peripheral blood CD14+ mononuclear cell population and not CD14- population; CD14 expression was lost during differentiation of fibrocytes (20, 32, 87). The ability to differentiate fibrocytes from the CD14+ fraction of PBMCs *in vitro* may indicate that fibrocytes are derived from monocytes, but another possibility is that monocytes and distinct fibrocyte precursors simply share surface expression of this marker. Absence of CD14 expression on fibrocytes might instead be better considered as an indication of the extent of fibrocyte differentiation. In addition, for cultured cells prolonged exposure to the artificial environment and high concentrations of growth factors present in tissue culture is known to cause genetic and epigenetic alterations may override inherent potential and cell-autonomous fate decisions, inflating differentiation potential (76). As applied to cultured PBMCs, a blood-derived cell that becomes a fibrocyte in culture may not have possessed the potential to do so in response to natural cues *in vivo*. Thus, a culture-enriched population of fibrocytes may misrepresent the origin and phenotype of the *in vivo* fibrocyte population.

Fibrocyte differentiation from monocytes and macrophages has been investigated *in vivo*. In a coronary artery ligation model of MI, *in situ* hybridization performed 7 days following ligation found 47% of *EMR1* (i.e., gene for F4/80) expressing macrophages also expressed *Col1a1* and 26% co-expressed *Col1a1* and the myofibroblast marker *Postn (* 82). One quarter of *EMR1*+ cells, expressed the monocyte marker *Ccr2* suggesting these macrophages were derived from newly infiltrated monocytes, however none of these monocyte-derived macrophages expressed *Col1a1* or *Postn*. This suggests that in this particular model if newly infiltrated monocytes differentiate into fibrocytes they must first pass through an intermediate phenotype (e.g., macrophage) with loss of *Ccr2* expression. A hematopoietic-derived *EMR1*- population was not characterized, therefore it cannot be concluded whether monocytes may differentiate into fibrocytes without transitioning through and *EMR1*+ macrophage-like intermediate. In addition, identifying macrophages by *EMR1* expression does not distinguish monocyte-derived macrophages from tissue resident macrophages, so this study was not designed to determine whether one of these populations might have a greater propensity for differentiating into fibrocytes.

The effect of depleting potential BM-derived myeloid precursors of fibrocytes has been investigated in a unilateral ureteral obstruction (UUO) model of kidney fibrosis performed in chimeric mice generated by transplanting BM from *CD11b-DTR* donors into lethally irradiated WT recipients (14). In these transgenic mice, expression of the diphtheria toxin receptor (DTR) is under the control of the CD11b promotor and administration of diphtheria toxin (DT) results in depletion of CD11b expressing cells. BM chimeras allow for selective depletion of *CD11b* expressing BM-derived cells and not other BM-derived cells or host cells that express *CD11b*. A single injection of DT reduced peripheral blood monocytes by ~50% and subsequent injections almost completely depleted monocytes in the blood and obstructed kidney. Following monocyte depletion, in the obstructed kidney Col1+ CD45+ fibrocytes were reduced compared to mice not treated with DT. One interpretation is that circulating fibrocytes and/or fibrocyte precursors were depleted, or that infiltration into the kidney had been impaired. However, CD11b is not specific for fibrocyte precursors and myeloid cells expressing CD11b are a source of cytokines and growth factors that may stimulate the differentiation of tissue resident macrophages into fibrocytes or the activation of tissue resident fibrocyte precursors. Thus, this model does not allow for determining whether fibrocytes originate from circulating BM-derived cells or from non-BM-derived fibrocyte precursors already present within the kidney.

The prior examples demonstrate that, by focusing on BM-derived cells, BM chimera and parabiosis models may fail to identify fibrocytes derived from hematopoietic-derived cells pre-existing within tissues and therefore provide an incomplete picture of the contribution of fibrocytes to fibrosis. Few studies address “*other tissue sources*”, as suggested as a possible origin in the study that first identified fibrocytes (4). It may be that having named these cells fibrocytes because they produce collagen (fibro) and may be derived from the circulation (cyte), in addition to the common practice of isolating fibrocytes from PBMCs, creates the potential to mistakenly interpret that all fibrocytes must be *recently* derived from the circulation. Hematopoietic-derived cells established early during development exist in tissues throughout the body with varying rates of replenishment from the BM (88). If one were to design studies based on a broader interpretation that includes hematopoietic-derived cells regardless of their timing or journey from genesis during hematopoiesis to presence within a tissue, one could achieve a more comprehensive understanding of the origin or origins of fibrocytes. As an example, one source of a potential fibrocytes that would be included under this broader interpretation of hematopoietic-derived cells are peritoneal macrophages that can produce collagen in culture (89, 90).

Perhaps contributing to this potential confusion is that cultured fibrocytes express CD34, also called HSC antigen. Expression of this hematopoietic marker with isolation of fibrocytes from PBMCs suggests that HSCs in the BM may be a source fibrocytes. What is then easy to overlook is that while BM could be “a” source, it is not necessarily “the” source of fibrocytes. CD34 expression has since been found in tissues outside of the BM and on cells that express other hematopoietic markers. Within subcutaneous adipose tissue, ~10% of uncultured, freshly isolated macrophages obtained from stromal vascular fraction of human lipoaspirates expressed CD34, CD45, and CD206 (91–93). These cells localized in the perivascular region and share characteristics with adipose-derived MSCs and circulating monocytes. In culture, they grew rapidly, after several weeks in 10% FBS lost expression of CD45, CD14, and CD206, and could also be differentiated into multiple mesenchymal lineages. Therefore, tissue resident macrophages are an abundant source of cells and it is plausible that these cells were the primary source of cells observed within the subcutaneous wound chamber in the original study that identified fibrocytes but failed to show fibrocytes were BM-derived when using chimeric mice that they were BM-derived.

CD34 is also expressed on hematopoietic macrophage progenitors that reside in the adventitia of blood vessels (31, 94). Within healthy WT mice, 8.4% of cells isolated from the aorta express CD34 compared to only 0.7% in the blood and 1.5% in the BM; although, not all adventitial CD34+ cells are of hematopoietic lineage as expression of CD34 is also found on adventitial mesenchymal progenitor cells that do not express hematopoietic markers (31, 95, 96). Following irradiation, the clonogenic capacity of these adventitial macrophage progenitors was reduced, but these cells were not replenished by circulating cells from the BM or spleen and they were not depleted by clodronate containing liposomes (94). Therefore, this potential source of fibrocytes would persist in chimeric mice following BM transplantation and since the vasculature is abundant in nearly all tissues these adventitial hematopoietic cells could be a local source of fibrocytes throughout the body that are not recently derived from the circulation.

The presence of fibrocytes within a tissue does not require an underlying pathology as fibrocytes have been identified in the heart, spleen, skin, and kidneys of healthy mice (9, 10, 14, 23, 97). scRNA-Seq performed on cardiac fibrocytes from healthy mice revealed these cells possess a hybrid phenotype between fibroblasts and macrophages, expressing intermediate levels of canonical macrophage genes (*Fcgr1*, *Cd14*, and *Ptprc*) and fibroblast genes (*Col1a1*, *Pdgfra*, and *Tcf21*) (97). In a separate study, flow cytometry was used to characterize tissue resident macrophages (CD45lo), monocyte-derived macrophages (CD45hi), and fibrocytes (Col1+ CD45+) (23). The number of monocyte-derived fibrocytes was greater in hearts from aged mice (20-30 month-old) as compared to young mice (3-month-old). In addition, median fluorescence intensity for Col1 was twice the value in aged hearts compared to young hearts. There was also a population of Col1+ tissue resident macrophages in the heart and this is significant because cardiac tissue resident macrophages arise from yolk sac and fetal monocyte progenitors (98). Yolk sac macrophages precede the formation of definitive hematopoiesis in the BM. Therefore, were any tissue resident yolk sac-derived macrophages to express collagen these would not fit the popular definition for a fibrocyte as a BM-derived cell, though this distinction may be more formal than practical (88). In addition to the heart, the brain and liver are among the few tissues known to retain yolk sac-derived macrophages (98).

The presence of fibrocytes in tissues of healthy animals raises the possibility of a tissue resident fibrocyte population and local proliferation as a potential source of new fibrocytes, but this is not supported by strong evidence. While PBMC-derived fibrocytes cultured *in vitro* display a doubling time of 3 to 4 days, proliferation *in vivo* has not been adequately investigated (4, 9). A study of fibrocytes in a UUO model of kidney fibrosis did not communicate an attempt to quantify proliferation of resident fibrocytes, but addressed this possibility in the discussion by stating “*so far, no proliferation of mature fibrocytes has been found*” (14). In a mouse model of asthma, when circulating Col1+ CD34+ cells were labeled with the fluorescent red dye PKH-26 and reinjected, after 24 hours the intensity of PKH-26 was slightly reduced indicating that a minority of the cells may have divided (9).

Col1+ CD45+ fibrocytes can also be found in the spleen of healthy individuals, the number of which exceeds the number fibrocytes isolated from blood by approximately an order of magnitude and the obstructed kidney in a UUO fibrosis model by approximately two orders of magnitude (9, 14, 19). Given the relative number of Col1+ fibrocytes that can be detected within and isolated from the spleen, similar to its known function as a reservoir for monocytes it is worth considering the spleen as a potential reservoir for fibrocytes (99). It remains to be determined whether splenic fibrocytes are readily deployable in response to inflammation or if the spleen functions as a central repository for aged fibrocytes and/or site at which they might undergo recycling similar to erythrocytes in the liver. Alternatively, the spleen may provide a site for fibrocyte differentiation, analogous to maturation of T cells in the thymus. While intriguing, this currently does not have strong support in the literature. Fibrocyte differentiation from monocytes in the spleen was investigated in a UUO kidney fibrosis model. Following obstruction of the kidney, there was an increase in the number of Col1+ CD45+ fibrocytes in the spleen (14). Depletion of monocytes using an antibody against CCR2 did not also result in depletion of fibrocytes in the blood or spleen indicating that fibrocytes may not directly differentiate from CCR2+ monocytes. The authors of the UUO kidney model concluded that differentiation of monocytes into fibrocytes within the spleen was unlikely in part because depletion of circulating monocytes did not result in a reduced number of fibrocytes in the spleen.

However, if monocytes do differentiate into fibrocytes, the lack of detectable fibrocyte depletion in the spleen following CCR2+ monocyte depletion has plausible explanations. First, the population of splenic fibrocytes is relatively large. If the rate of fibrocyte differentiation was low and splenic fibrocytes were to possess a relatively long lifespan compared to monocytes, a decrease in splenic fibrocytes due to monocyte depletion or inhibited monocyte infiltration into the spleen might simply be difficult to detect. A second possible explanation requires a bit of background. In mice, two subpopulations of monocytes have been identified, namely classical monocytes (Ly6C^hi^ CCR2+) and non-classical monocytes (Ly6C^lo^ CCR2-) (100). In humans, similar subpopulations exist that include classical (CD14+ CD16-), intermediate (CD14+ CD16+), and non-classical (CD14^lo^ CD16+) monocytes. Restoration of circulating monocytes following monocytopenia demonstrated sequential transition of monocytes from classical through intermediate to non-classical phenotypes with lifespans of ~1, ~4, and ~7 days, respectively (101). Classical monocytes are relatively proliferative and resistant to apoptosis compared to non-classical monocytes which appear relatively senescent and are proposed to be terminal differentiated (102). Associated with the differences in surface marker expression are differences in function. Classical monocytes are regarded as pro-inflammatory and migration out of the BM and into sites of inflammation is CCR2-dependent (103). Non-classical monocytes are termed patrolling as they crawl along the lumen of the vasculature. After extravasating into tissue, classical monocytes may give rise to non-classical monocytes, macrophages (both CD206+ and CD206-) and dendritic cells, whereas non-classical monocytes preferentially give rise to CD206+ macrophages (103–105). Returning now to the second potential explanation, if fibrocytes differentiate preferentially from non-classical monocytes within the spleen, one might expect a delay for the depletion of classical monocytes to result in a depletion of non-classical monocytes and subsequent reduction in fibrocyte differentiation such that the effect was not detectable within the 7 days during which this experiment was performed. In other words, the treatment may not have had a direct or immediate effect on the pool of cells actively differentiating into fibrocytes and adequate numbers of these monocyte-fibrocyte intermediates were maintained such that fibrocyte generation was not adversely impacted. Therefore, the lack of detectable change in splenic fibrocyte number following antibody depletion of CCR2+ monocytes in the UUO model suggests that if fibrocytes differentiate from monocytes within the spleen, fibrocytes do not appear to differentiate directly from classical CCR2+ monocytes. Correspondingly, Col1+ cells expressing classical monocyte markers were not observed in the spleen. Splenic fibrocytes may instead differentiate from non-classical CCR2- monocytes, splenic macrophages, or infiltrate as differentiated fibrocytes.

The possibility that fibrocytes might differentiate within the spleen warrants further study. One strategy could be to splenectomize mice and determining if there a subsequent reduction in the number of circulating fibrocytes and/or fibrocytes within tissues following injury. This would rest on the assumption that no compensatory mechanism exists such as alternative sites of fibrocyte differentiation.

Col1+ hematopoietic-derived cells in the circulation, BM, and spleen have largely been interpreted to be newly-derived fibrocytes. Another possibility is that these cells may instead be derived from tissue fibrocytes or Col1+ macrophages that have reentered the circulation allowing them to traffic to the BM and spleen. Regarding Col1+ macrophages, tissue resident macrophages are established during development and these may be supplemented by monocyte-derived macrophages that differentiate after entering a particular tissue. Therefore, one might not expect to observe macrophages within the blood. Yet, in healthy humans 2.6% of PBMCs expressed the macrophage marker PM-2K (106) and in healthy mice between 0.5 and 1% of PBMCs expressed F4/80, a similar value to the frequency of fibrocytes reported in the blood (107). In an attempt to explain this phenomenon, macrophage-like cells have been observed in the thoracic duct, which is a terminal lymphatic vessel that drains into the systemic circulation, and macrophages bearing specific tumor or tissue antigens have been detected within the circulation (108–110). In mice, 24 hours following injection of the lipophilic membrane dye dioctadecyloxacarbocyanine (DiO) into the myocardium ~2% of macrophages in the spleen and ~5% of macrophages in the BM were DiO+, values that increased if mice were subjected to coronary artery ligation immediately following injection of DiO (111). These observations appear to confirm that Col1+ macrophages may return to the circulation either directly or indirectly through the lymphatic system where they may then traffic to the BM and spleen (112). Perhaps fibrocytes within tissues may recirculate and traffic by similar mechanisms. Therefore, as an alternative to increased generation or recruitment from the BM, fibrocyte and Col1+ macrophage reentry into the circulation could explain the increased number of fibrocytes observed in the spleen following injury to the kidney, liver, and skin (10, 14, 113, 114).

In the original study that identified fibrocytes, the authors suggested BM-MSCs might be a potential fibrocyte precursors. This now appears highly unlikely since BM-MSCs do not circulate and should not contribute to fibrosis outside of the BM, with the exception of severe bone fracture and BM embolism (29, 66). In addition, CD45 is an exclusion criterion for MSCs. Although MSCs have been reported to express macrophage markers, acquired expression of CD45 has not been reported on these cells.

**Figure 2:**
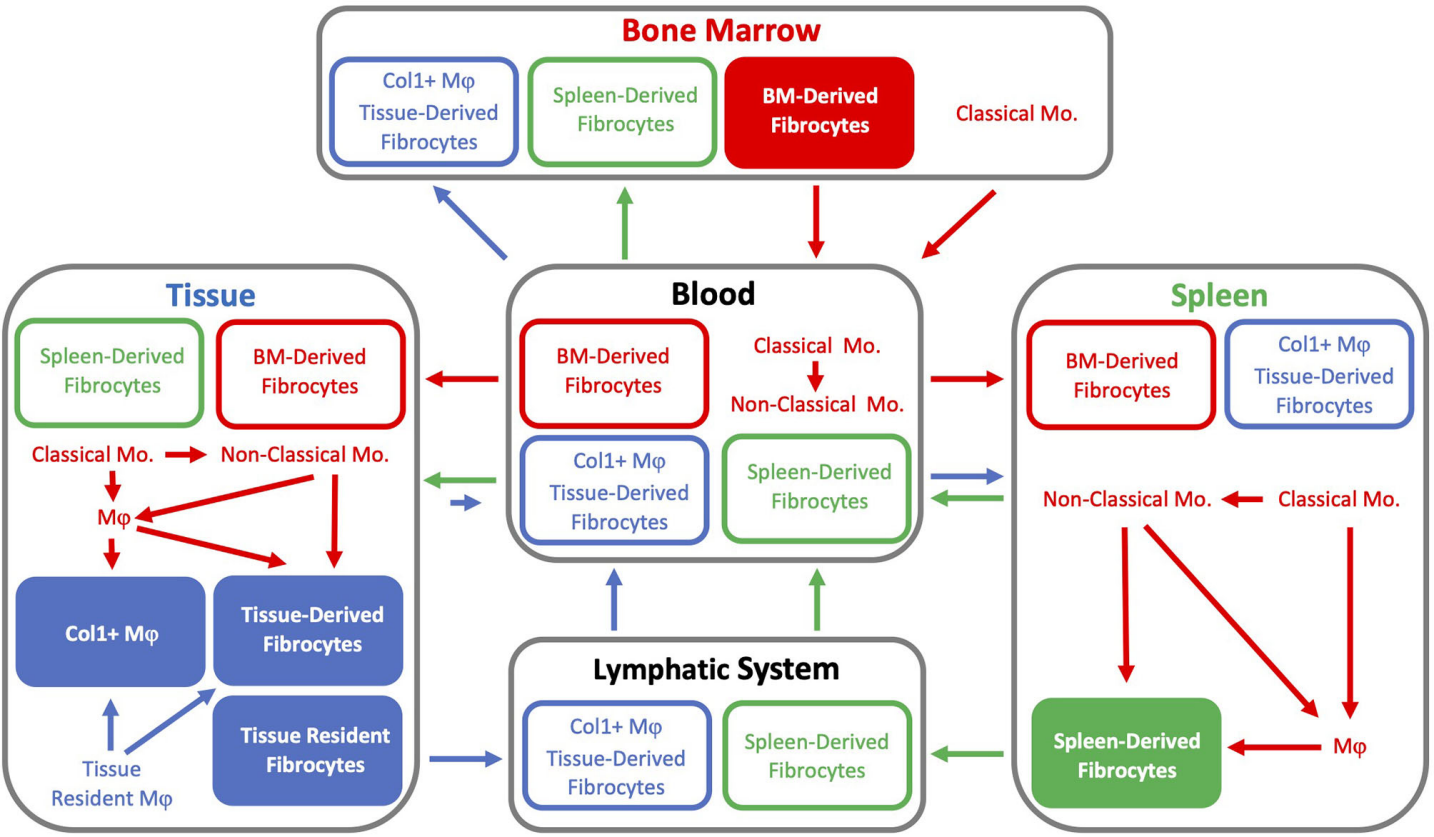
Potential origins and trafficking of fibrocytes. This diagram shows potential fibrocyte precursors and sites of differentiation. In addition, it shows the trafficking of fibrocytes as well as Col1+ macrophages that do not express collagen that could account for the identification of Col1+ hematopoietic-derived cells within the bone marrow, blood, spleen, and in peripheral tissues. Fibrocytes may differentiate from a dedicated precursor within the bone marrow before being released into the circulation. Alternatively, fibrocytes may instead differentiate from monocytes or macrophages. Classical monocytes are continuously generated by hematopoiesis in the bone marrow and are released into the circulation. Most monocytes leave the circulation to enter a particular tissue as classical monocytes, but some are retained and differentiate into non-classical monocytes. While limited evidence suggests that classical monocytes do not directly differentiate into fibrocytes, non-classical monocytes, monocyte-derived macrophages, and tissue resident macrophages may be possible fibrocyte precursors. In addition, like tissue resident macrophages there may also exist a tissue resident population of fibrocytes established early in development. Both fibrocytes and Col1+ macrophages may reenter the circulation through the lymphatic system allowing them to traffic to the spleen or bone marrow. Differentiation of monocytes and macrophages into fibrocytes may also occur in the spleen and/or splenic fibrocytes may have extra-splenic origins. Potential origins of fibrocytes in the bone marrow (red), tissue (blue), and spleen (green) are identified by the solid-colored bubbles. The arrows are color-coded to identify the directional trafficking of each population and the potential for each cell population to be present outside of their anatomical compartment of origin is indicated by bubbles outlined in the color according to the location of origin. For example, spleen-derived fibrocytes may reenter the circulation directly or indirectly through the lymphatic system. Once in the circulation, spleen-derived fibrocytes may then traffic to the bone marrow or another tissue. bone marrow (BM), Col1 immunolabeled (Col1+), monocyte (Mo.), macrophage (Mφ), peripheral blood mononuclear cells (PBMCs).

Currently, it appears that the origin(s) of fibrocytes and sites of fibrocyte differentiation remain undetermined. Fibrocytes may be minted as a unique lineage within the BM, differentiate from monocytes or tissue resident macrophages, or arise from tissue resident fibrocyte precursors established early during development (**Figure 2**). Fibrocyte differentiation from precursors may occur within the BM, spleen, or after infiltrating into a particular tissue. Though, if monocytes do differentiate into fibrocytes, classical monocytes appear to require differentiation through an intermediate phenotype such as a non-classical monocyte or macrophage prior to the expression of collagen. Furthermore, fibrocytes may be derived from combinations of these potential sources and the relative contributions of these sources may be tissue and model specific.

## 3 Discussion

After more than 25 years of investigation, the direct contribution of fibrocytes to fibrosis has been reported in tissues throughout the body, yet arguably we still know little about these cells. In particular, there is a lot of uncertainty surrounding the life cycle of these cells. Though by definition these cell must have a hematopoietic origin, the identity of fibrocyte precursors has yet to be determined. In addition, the fate of these cells is unknown after the stimulus for fibrocyte recruitment and differentiation within a tissue resolve. Do fibrocytes undergo apoptosis, dedifferentiate, or migrate out of the tissue and reenter the circulation to traffic to another site such as the BM or spleen? If fibrocytes do dedifferentiate, for example into a macrophage-like cell, would that diminish the status of fibrocytes as a unique cell type and suggest collagen production is within phenotypic potential of hematopoietic-derived cells that is brought out by certain pathologies, aging, or artificial environments? Most of the fibrosis models provided are of short duration from 1-6 weeks after injury to the time of analysis. In resolving wounds, such as the skin, the presence of fibrocytes decreased after peaking at one week (7). Few studies have investigated the long-term effect of fibrocytes as might be expected as part of a foreign body response surrounding an implant (115). It would be interesting to determine the lifespan of fibrocytes within a particular setting, whether there is continued accumulation throughout chronic inflammation, and whether extended exposure to an inflammatory environment might result in heightened ECM production or increased contractile potential compared to what has been observed acutely.

The combination of stimuli necessary to elicit collagen expression from fibrocyte precursors remains to be determined. Similar to myofibroblast differentiation, TGF-β1 may be one such stimulus. TGF-β1 supplementation increased the expression of Col1 and αSMA in cultured BM-derived CD11b+ cells (10). Since this measurement was determined by qPCR performed on pooled cells, it can’t be known whether TGF-β1 increased the fraction of cells expressing these markers, or increased the expression of these markers only in cells already expressing Col1 and αSMA. TGF-β1 also increased the expression of αSMA 4-fold in cultured human PBMCs as well as their ability to contract collagen gel (20, 22). Similarly, another study reported that in cultures of Col1+ CD34+ cells isolated from the peripheral blood, after 6 days in serum free medium 20% expressed αSMA, but more than 80% expressed αSMA in the presence of TGF-β1 (9).

It may also be worth revisiting the criteria for identifying fibrocytes. When fibrocytes were first described, these cells were shown to express Col1 and Col3. Subsequent studies have largely relied on expression of Col1 for fibrocyte identification, but transcriptional analyses have shown that fibrocytes may also produce Col4, Col5, Col6, Col8, Col12 fibrillin, periostin, and biglycan (5, 17). A consensus should be established as to whether there exist other ECM proteins for which expression in the absence of Col1 expression would be sufficient for a hematopoietic-derived cell to be considered a fibrocyte.

Recent transcriptional analyses based on scRNA-Seq data have demonstrated the potential to vastly expand the ability to identify and characterize fibrocytes *in vivo* with increased confidence. Though scRNA-Seq is powerful on its own, its future application will likely complement many of the methods described herein including transgenic mice and histology; hence, the motivation for revisiting the strengths and limitations of these strategies for studying fibrocytes. The investigative power of methods like scRNA-Seq may also justify revisiting past experiments limited by the methods applied at the time. In conclusion, the future application of single-cell transcriptional analyses promises to invigorate the study of fibrocytes and accelerate our understanding of this cell type.

## Author Contributions

JR and CB conceived and designed the manuscript. JR conducted the literature review and drafted the manuscript. JR and CB critically revised the manuscript. All authors contributed to the article and approved the submitted version.

## Funding

This research was supported by U.S. National Institutes of Health grants: R01HL098228, R01HL128602, and R01HL128847, in addition to the Department of Defense Application ID PR170976 Award Number W81XWH-18-1-0518. JR was supported by the American Heart Association under Award Number 18POST33990231.

## Conflict of Interest

The authors declare that the research was conducted in the absence of any commercial or financial relationships that could be construed as a potential conflict of interest.

## Publisher’s Note

All claims expressed in this article are solely those of the authors and do not necessarily represent those of their affiliated organizations, or those of the publisher, the editors and the reviewers. Any product that may be evaluated in this article, or claim that may be made by its manufacturer, is not guaranteed or endorsed by the publisher.

## Abbreviations

BM, Bone marrow; Col1a1, procollagen type I α1; Col1a2, procollagen type I α2; DT, diphtheria toxin; HSC, hematopoietic stem cell; MSC, mesenchymal stem cell; PBMC, peripheral blood mononuclear cell; PICP, procollagen type I C-terminal propeptide; PINP, procollagen type I N-terminal propeptide; scRNA-Seq, single-cell RNA-Sequencing; UUO, unilateral ureteral obstruction.
